# Investigation of Self-Assembly and Charge-Transport Property of One-dimensional PDI_8_-CN_2_ Nanowires by Solvent-Vapor Annealing

**DOI:** 10.3390/ma12030438

**Published:** 2019-01-31

**Authors:** Haixiao Xu, Jianqun Jin, Jing Zhang, Peng Sheng, Yu Li, Mingdong Yi, Wei Huang

**Affiliations:** 1 Key Laboratory for Organic Electronics and Information Displays & Jiangsu Key Laboratory for Biosensors, Institute of Advanced Materials (IAM), Jiangsu National Synergetic Innovation Center for Advanced Materials, Nanjing University of Posts & Telecommunications, 9 Wenyuan Road, Nanjing 210023, China; iamhaixiaoxu@163.com (H.X.); jqjin2017@163.com (J.J.); muziyu1995@126.com (Y.L.); iammdyi@njupt.edu.cn (M.Y.); 2 Material Laboratory of State Grid Corporation of China, State Key laboratory of Advanced Transmission Technology, Global Energy Interconnection Research Institute, Beijing 102211, China; shpjob@163.com; 3 Shaanxi Institute of Flexible Electronics (SIFE), Northwestern Polytechnical University (NPU), 127 West Youyi Road, Xi’an 710072, Shaanxi, China

**Keywords:** solvent-vapor annealing, PDI derivative, long-range and entire transport, 1D nanowires, morphology tailoring, electron transport

## Abstract

One-dimensional (1D) nanowires have attracted great interest, while air-stable *n*-type 1D nanowires still remain scarce. Herein, we present solvent-vapor annealing (SVA) made nanowires based on perylene tetracarboxylic diimide (PDI) derivative. It was found that the spin-coated thin films reorganized into nanowires distributed all over the substrate, as a result of the following solvent-vapor annealing effect. Cooperating with the atomic force microscopy and fluorescence microscopy characterization, the PDI_8_-CN_2_ molecules were supposed to conduct a long-range and entire transport to form the 1D nanowires through the SVA process, which may guarantee its potential morphology tailoring ability. In addition, the nanowire-based transistors displayed air stable electron mobility reaching to 0.15 cm^2^ V^−1^ s^−1^, attributing to effective in situ reassembly. Owing to the broader application of organic small-molecule nanowires, this work opens up an attractive approach for exploring new high-performance micro- and nanoelectronics.

## 1. Introduction

One-dimensional (1D) nanowires have attracted extensive attention owing to their potential application in constructing optoelectronic devices [1,2,3], for instance, organic field-effect transistors [4], sensors [5,6,7], nanogenerator [8,9], thin-film solar cells [10] and photowaveguide materials [11]. Inorganic nanowire thin films, such as Ag [12] and TiO_2_ nanowire network [13] via solution-processed method have been introduced to act as transparent electrodes to reproduce the performance of common electrodes on various substrates. In contrast to the inorganic electronic materials, organic conjugated nanowires or nanowire networks presented improved mechanical properties of flexible and stretchable ability, especially for organic conjugated polymers [14,15,16] including conducting and semiconducting materials. The superior electrical performance [17] and low-cost direct printing technique [18,19,20] even enable their further use in next generation devices for wearable electronics and implantable biomedical applications. Different from polymer, small molecules are easy to synthesize and purify, readily accessible in large quantities, can be deposited by solution and evaporation techniques, and have produced the highest mobilities reported to date [21]. But for the majority of organic small molecules, low molecule weight, low viscosity and relative smaller solubility restricted the one-dimensional nanowire preparation method. Their high structural tunability, reaction activity and processability provide great opportunities to miniaturized optoelectronic chips based on organic 1D nanostructures, since they are usually assembled from molecular units with weak intermolecular interactions, such as hydrogen bonds, *π-π* stacking and van der Waals force. These weak interactions allow for more facile and mild conditions in the fabrication of high quality organic 1D nanostructures rather than those in the construction of their inorganic counterparts [11]. At first, solution-processed small-molecule nanowires (NWs) are grown in situ on the substrates through drop-casting approach [22], as the self-assemble [23,24] process occurs with solvent evaporation and molecules align to the preferred direction, or dip-coating method [25,26] by vertically pulling the substrate out of an organic solution, then other essential components will be applied to the target active nanowires to form functioning electronic devices. For example, Frederick and his co-workers doped F_4_-TCNQ (*p*-dopant) into P3HT in the solution phase, which showed higher aggregation rate of 1D P3HT nanowires with smoother edges and less protruding segments [15]. Through researchers’ attempt to obtain the nanowires of organic small semiconductors to develop the new generation strategy, such as hierarchy patterning on solid substrates [27], using polymer as a soluble crystal modifier [28], there has been some progresses in tailoring nanocrystalline morphologies of organic semiconductors (OSCs).

However, the progress achieved with *p*-type nanowires OSCs outperforms that realized with *n*-type materials. This is mainly due to the lower stability of the radical anions formed upon injection of electrons into the OSC [29]. Oxygen or water molecules penetrating into the OSC layer can easily oxidize the radical anions. Perylene tetracarboxylic diimides derivatives (PDI derivatives) as typical *n*-type OSCs have been widely investigated since first reported by Horowitz et al. [30], followed by N-substituted alkyl chain substituted ones [31]. Fluorinated [32] and 1,7-Dicyano [33] derivatives of PDIs usually exhibits excellent air stability and good performance owing to the reduced the lowest unoccupied molecular orbitals (LUMO) energy level. Among these materials, N,N’-bis(n-octyl)-(1,7&1,6)-dicyanoperylene-3,4:9,10-bis (dicarboximide) (PDI_8_-CN_2_) is one of the most widely used solution processible PDI derivatives OSCs [34,35]. Salleo and co-workers prepared the aligned PDI_8_-CN_2_ film by solution-cast method to reveal the effects of grain-boundary type and trapping barriers, which showed strong charge-transport anisotropy with mobilities for parallel devices reach 10^−2^ cm^2^ V^−1^ s^−1^ [36].

**Figure 1:**
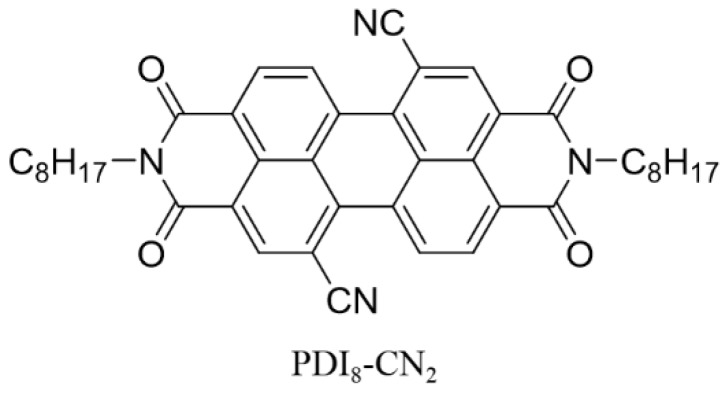
Chemical structure of PDI_8_-CN_2_.

In this work, we report use of solvent-vapor annealing (SVA), for fabrication of nanowires of PDI_8_-CN_2_ (as shown in Figure 1) on various substrate surfaces, to fabricate air stable *n*-type transistors. Solvent-vapor annealing was performed on a dielectric surface, with solvent vapor to dissolve the organic materials which then reorganize into a higher degree of order to form functional nanostructures [37,38]. The nanowires exhibits a moderately electron transport up to 0.15 cm^2^ V^−1^ s^−1^, superior air stability and on/off ratio, which can be comparable to the single-crystal devices (0.183 cm^2^ V^−1^ s^−1^). These findings suggest solvent-vapor annealing can be an efficient strategy to form high-performance *n*-type nanowires.

## 2. Results and Discussion

**Figure 2:**
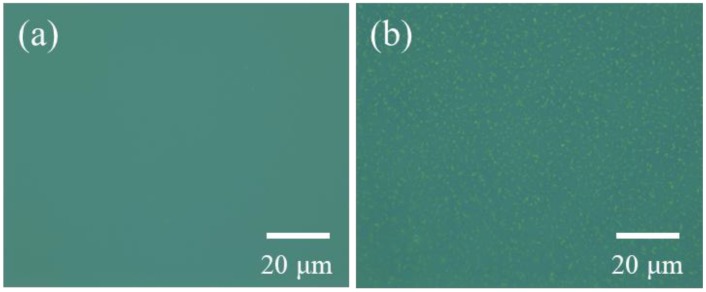
Optical microscope images of spin-coated nanostructures with 1:1 mass ratios of PDI_8_-CN_2_/PMMA at a total concentration of 5mg/mL on a Si/SiO_2_ substrate (**a**) before and (**b**) after SVA.

Poly(methyl methacrylate) (PMMA) usually acted as a passivation layer in electronic devices, leading to the optimized organic active semiconductor/inorganic insulator interface. Herein, we conducted a self-organized phase separation. Two components mixed in solution automatically separated during spin-coating and form a bilayer-structured film. PDI_8_-CN_2_ and PMMA were mixed in 1,2-dichlorobenzene with different mass ratios, then spin-coated the mixture onto Si/SiO_2_ substrates to form the active semiconductor layer on top of an amorphous layer. Optical microscope images of micro/nanostructures of different mass ratios of PDI_8_-CN_2_/PMMA at a total concentration of 5 mg/mL before and after solvent-vapor annealing treatment (SVA) were then investigated. A flat thin film without obvious large grains was observed from the 1:1 ratio as shown in Figure 2a. After the SVA process, the former structures aggregated into small sized clusters (Figure 2b), indicating no sufficient reaction to the annealing of this 1:1 mixed preparing condition.

**Figure 3:**
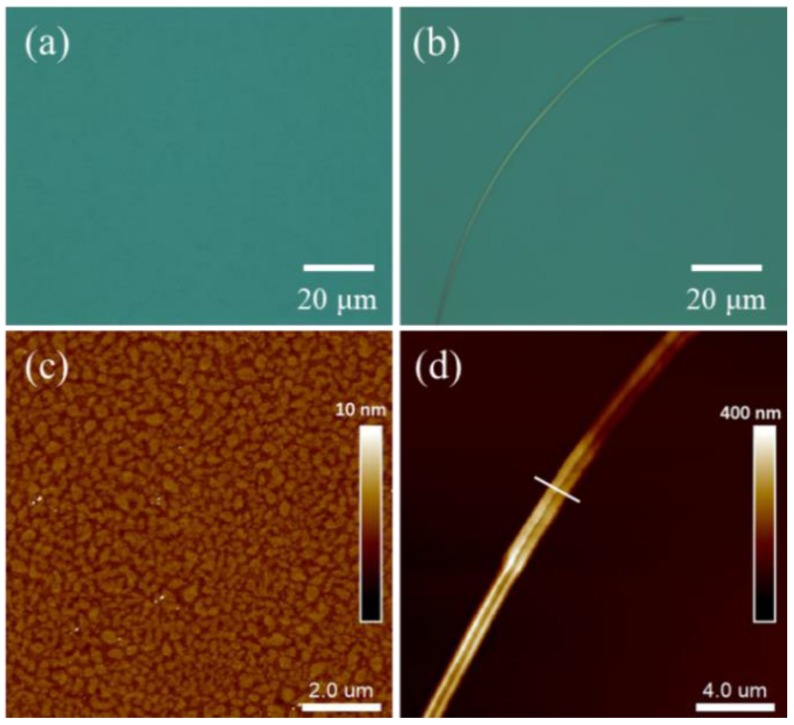
(**a**,**b**) Optical microscope images and (**c**,**d**) AFM images of spin-coated nanostructures with 1:20 mass ratios of PDI_8_-CN_2_/ PMMA at a total concentration of 5mg/mL on Si/SiO_2_ substrate (**a**,**c**) before and (**b**,**d**) after the SVA process.

When the PDI_8_-CN_2_ concentration was narrowed to be 0.3–0.5 mg/mL (the mass ratio was 1:10 or 1:20), it came to the similar result, so we take the 1:20 as the example. As shown in Figure 3a, the as-prepared thin film seemed to be flat and no obvious clusters existed. According to the atomic force microscopy (AFM) image (Figure 3c), the thin fil m was comprised of discontinuous nanoflakes, with almost the same height and size of 300–500 nm. The flake height was about 3.0 nm, while the length of PDI_8_-CN_2_ molecule is ~3.1 nm based on density function theory (DFT) calculation, which implied that the mono-layer molecules stood on the substrate with a tilt angle, and the average roughness (Rq) of the nanostructure was calculated to be ~0.59 nm. Compared with the blend films, the AFM images of pure PDI_8_-CN_2_ clusters spin-coated from 1,2-dichlorobenzene solution of 5 mg/mL showed a larger average height of about 10.2 nm and an average roughness of ~3.28 nm (Figure S1). Obviously, after the introduction of PMMA, the PDI_8_-CN_2_ was then well-distributed, the aggregation was greatly reduced, and the roughness was substantially decreased, which was conducive to the long-distance mass transfer of PDI_8_-CN_2_ molecules during the following SVA process. In addition, PMMA improved the interface quality because of its hydrophilicity, lower interface energy and polarity. Self-assembled nanowires were obtained all over the substrate after SVA process (Figure 3b), with the length up to hundreds of micrometers, the width of hundreds of nanometers to several micrometers (Figure 3d). Besides, the height of the nanowire varied from one hundred nanometers to several micrometers, 1–2 orders of magnitude larger than the clusters in original blend films (Figure S2). From the above results, we believed that the concentration of PDI_8_-CN_2_ component is one of the essential factors to determine the semiconductor topography during and after the SVA approach. When the concentration of PDI_8_-CN_2_ was lower (such as 1:50 *w*/*w* ratio), less nanostructures emerged as we supposed, the length was reduced to no more than 100 µm and width was increased to several to ten micrometers, compared to the 1:20 situation, meanwhile multiple nanostructures bunched together (Figure S3). This phenomenon may be due to the fact that the low concentration of PDI_8_-CN_2_ induced a larger neat surface and faster dissolution, obstructing well-distribution and long-size self-assembly of PDI_8_-CN_2_ molecules spread on the PMMA surface. The above results revealed that the self-assembly can be controlled by tailoring the concentration of PDI_8_-CN_2_, so as to adjust the nanowires morphology after annealing which including length, cross-section and thickness. 

The XRD pattern of the PDI_8_-CN_2_ nanowires from 1:20 PDI_8_-CN_2_/PMMA mixed solution showed a clear peak in 4.4° with a d-spacing of 20.1 Å (Figure S4), corresponding to the c-axis of the PDI_8_-CN_2_ unit cell, and therefore attributed to (001) reflections of the predicted crystal structure [39,40]. The self-assembly driven by *π-π* interactions enabled the growth direction along the b direction between neighboring PDI cores [41].

Solvent-vapor annealing method [42,43] employs saturated solvent vapor to dissolve functional materials, then the active molecules reorganize into a higher degree of order [44]. This method is a useful strategy for the researchers to reach desired micro/nanostructures for organic nanoelectronics [45]. For the dissolution and molecule transport process, the solvent is another main factor. The choice of solvent is not limited to that used for the deposition from solution and the possibility to modulate the solvent vapor pressure, temperature, exposure time allows a high control over the adsorbate reorganization and the surface properties of the substrate. The surface-assisted solvent-vapor annealing was performed in a closed chamber saturated with appropriate solvent vapor. Factors that influence solvent choice include: (1) solubility in order to allow free molecules to be transported with the solvent on the surface; (2) minimum affinity to the surface, thus allowing high mobility on the surface. By mastering the solubility properties of the different materials in a chosen solvent, it is possible to modulate the degree of interaction between molecule-molecule, molecule-substrate, molecule-solvent and solvent-substrate. As a good solvent for PDI_8_-CN_2_, 1,2-dichlorobenzene with high boiling point was chosen as the typical solvent to carry out the vapor annealing of the sample cast on the surface of the substrate. The hydrophobic characteristic should enable effective molecular dynamics (and thus the packing) on the PMMA surface. According to the comparison between Figure S1 and Figure 3c, it is obvious that PMMA helped the spreading and homogenization of PDI_8_-CN_2_. PDI_8_-CN_2_ molecules tend to be well spreading due to the organic (PMMA) surface. Meanwhile, the mesoscopic phase separation distinctly occurred in the blend films and manifests itself in the protruded structure coexisting with another surrounding matrix. It is a direct consequence of the interplay between liquid-liquid mix and stratification during the spin-coating process [46,47,48]. In particular, the 1:20 blend film composes of domains (0.1–0.3 μm in diameter) randomly interspersed in the matrix [48,49,50,51]. When the bilayer architecture was exposed to 1,2-dichloroethane vapor, solvent vapors interacted with the substrate and, with the resolubilized adsorbate, promoted molecule transport mobility and rearrangement on the surface both at the molecular and the mesoscopic scale [52]. Therefore, PDI_8_-CN_2_ molecules started to crystallize at the air-film interface and after sufficient time (ca. 10 h) they reassembled into large-size nanowires. Such a solvent-vapor annealing process takes advantages of both the slow crystallization process (taking place in the minimal amount of solvent condensed on the surface) and the flat substrate (which confines the random walk of molecules from 3D in solutions to 2D on a surface), and thus facilitates the spatial organization of the molecules. Therefore, we believed that by mastering weak intermolecular interactions among PDI_8_-CN_2_ molecules through vapor annealing, it was possible to grow self-assembled nanowires with hundreds of micrometers long. Other common solvents failed to this SVA treat of this system, which proved our theory.

**Figure 4:**
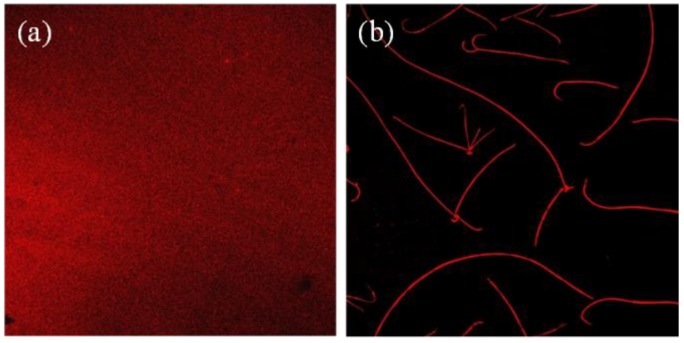
Confocal fluorescence microscope images of spin-coated thin film from PDI_8_-CN_2_/PMMA (1:20 *w*/*w* ratio) 1,2-dichlorobenzene solution on Si/SiO_2_ substrate before (**a**) and after (**b**) SVA process. Excitation wavelength was ~559 nm, all peaks of their fluorescence spectra ranged from ~655 nm to ~755 nm, with red emission.

The absorption spectrum of PDI_8_-CN_2_ in 1,2-dichlorobenzene solution showed well-structured vibrational bands at λ = 463, 492, and 529 nm (Figure S5). Compared with that of solution, the absorption spectra of nanowire demonstrated obvious red shift with peaks at 475, 510, and 554 nm, indicating strong π-π interaction in the solid state. The confocal fluorescence microscope images of spin-coated thin film nanostructures from PDI_8_-CN_2_/PMMA (1:20 *w*/*w* ratio) 1,2-dichlorobenzene solution on Si/SiO_2_ substrate before and after SVA were then analyzed. The analysis revealed that even the ultrathin nanoflake made film was observed to display strong and uniform fluorescence before the following treatment (Figure 4a). However, after the SVA process, only the as-prepared nanowires had strong fluorescence emission, no signs of fluorescence were found on other uncovered surface area, as shown in Figure 4b, indicating that the functional molecule had reached entirely long-distance transport. In contrast, PDI_8_-CN_2_ thin film by spin-coating 0.3 mg/mL 1,2-dichlorobenzene solution on the substrate resulted in irregular and scattered distribution of fluorescence due to the lack of PMMA modified layer (Figure S6). It could be concluded that PDI_8_-CN_2_ molecules were well-distributed on the surface for the addition of PMMA. This observation may be due to the high polarity of bare SiO_2_ surface, and then resulted in the aggregation of organic molecules which further influence the distribution of the reassembly on Si/SiO_2_ substrate.

In addition, PDI_8_-CN_2_ system without any polymer addition operated on Si/SiO_2_ substrate was also investigated (Figure S7). When the concentration of PDI_8_-CN_2_ component was 0.3 mg/mL, which was considered as the optimum concentration for nanowires growth, large-size nanowires were observed after the SVA process. As the concentration of PDI_8_-CN_2_ increased to 1 mg/mL, a small number of micro/nanowires appeared, with lots of aggregating grains. Additionally, when it came to 2 mg/mL, the size of the nanostructures drastically decreased, with the obvious increase in thickness, and almost all needle-like aggregates gathered in confined areas, which may be accounting to the large concentration. Enough molecules meet nearby and tend to cluster by the strong *π-π* interaction, making PDI_8_-CN_2_ molecules much shorter mass transport distance. This suggested that when the concentration of the original solution was ~0.3 mg/mL, the synthesized nanowires were of large-size length (several hundreds of micrometers) and ideal quantities. All the above results indicated adjusting the surface, choosing suitable solvent and tuning PDI_8_-CN_2_ concentration were the determining procedures to the nanowire growth of further nanoeletronic device fabrication.

**Figure 5:**
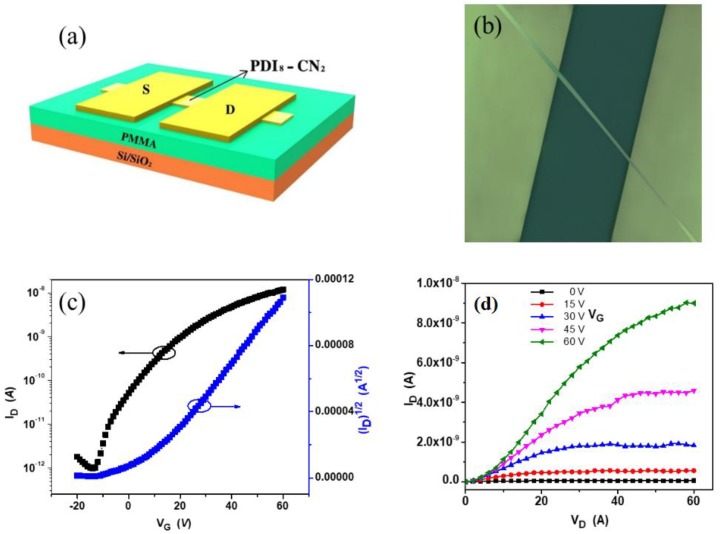
(**a**) Schematic diagram and (**b**) optical image of the device with an individual nanowire. (**c**) Transfer and (**d**) output characteristics (channel length, *L* = 24 µm; channel width, *W* = 0.6 µm) of the nanowire-based transistor.

In order to investigate the electrical properties of PDI_8_-CN_2_ nanowires, we constructed the nanowire-based *n*-type transistors with the bottom-gate top-contact geometry (Figure 5a). Au source/drain electrodes were then thermally evaporated onto the nanowires via a copper grid as the mask. Figure 5b shows the illustration of a single nanowire transistor. All the characterizations were conducted in air condition. Typical transfer and output characteristics are shown in Figure 5c,d, which showed well-saturated performance. Over 20 individual devices were measured and they exhibited an average *µ*_e_ (Electron Mobility) of 0.08 cm^2^ V^−1^ s^−1^ and *V*_th_ (Threshold Voltage) of 9 V. The highest electron transport mobility could reach to 0.15 cm^2^ V^−1^ s^−1^, with a relatively high on/off ratio of 10^5^. This result was comparable to the single-crystal devices, and better performance than the thin-film transistors [29,34,40]. Generally, the *π*-conjugated semiconducting materials tend to arranged along the *π-π* direction. The strong *π-π* interactions promote the PDI derivative movement during the SVA process, and facilitate the electron transport along the 1D direction. Besides, the devices showed nearly no degradation when measured after a month in air. We believed that the good air stability was attributed to the presence of the electron-withdrawing cyan (CN) moieties which were demonstrated to significantly lower the lowest unoccupied molecular orbital (LUMO). As is well known, lower LUMO lead the *n*-type semiconductors less susceptible to ambient atmosphere.

## 3. Experimental Section

### 3.1. Materials

*N,N’*-bis(*n*-ctyl)-*x:y*-dicyanoperylene-3,4:9,10-bis-(dicarboximide) (PDI_8_-CN_2_) was purchased from Polyera Corporation (Skokie, IL, USA), poly(methyl methacrylate) (PMMA) was obtained from TCI (Tokyo, Japan) and 1,2-dichlorobenzene was acquired from J&K Scientific (San Jose, CA, USA). All materials were used directly without further purification.

### 3.2. Nanowire Preparation

PDI_8_-CN_2_ and PMMA with a total concentration of 5 mg/mL were dissolved in 1,2-dichlorobenzene at a mass ratio of 1:1,1:10, 1:20, and 1:50, respectively. In addition, solutions of pure PDI_8_-CN_2_ dissolved in 1,2-dichlorobenzene at concentrations of 0.4, 1, 2 and 5 mg/mL were also prepared. All solutions were sonicated for 1 h and then heated at 120 °C until all components were dissolved completely. The solutions were spin-coated onto the substrates at a speed of 2000 rpm for 1 min in a nitrogen-filled glovebox. Then about 200 μL 1,2-dichlorobenzene was dropped in the covered glass dish, with the substrate inside, to anneal spin-coated films. Ultimately, sub-millimetric nanowires were observed on the substrate that followed. It is worth noting that 1,2-dichlorobenzene volatilizes slowly at room temperature, which may affect the self-assembly process, so the glass dish was heated until hot 1,2-dichlorobenzene vapor formed during the SVA process. 

### 3.3. Device Fabrication

Nanowire-based *n*-type organic field-effect transistors (OFETs) with a bottom-gate top-contact (BGTC) configuration were fabricated via thermally evaporating gold electrodes (0.3 Å/s, 7 × 10^−4^ Pa) through a copper grid, with a channel length (*L*) of ~20 μm.

### 3.4. Characterization

The as-prepared nanowires were characterized by Laser confocal fluorescence microscope (LCFM, Olympus FV1000MPE, Olympus Corporation, Tokyo, Japan), atomic force microscopy (AFM, Bruker Dimension Icon, Karlsruhe, Germany), Optical microscope (OM, Olympus BX3M-KMA-S, Olympus Corporation, Tokyo, Japan) and Powder X-ray diffraction (XRD, Rigaku D/Max2500/PC, Rigaku Corporation, Akishima, Japan). The electrical characteristics of the devices were measured with a Keithley 4200 SCS semiconductor parameter analyzer (Tektronix, Johnston, OH, USA) under ambient conditions. The mobility was evaluated in the saturated region.

## 4. Conclusions

In summary, sub-millimeter sized reorganized nanowires of an organic PDI derivatives (PDI_8_-CN_2_) compound was synthesized from in situ solvent-vapor annealing treatment. Upon exposure to solvent vapors, organic mono-layer was supposed to self-assembly into one-dimensional nanowires on the surface. Such 1D nanostructure growth was found to be a long-range and complete mass transport process (molecule movement) through the further investigation. They exhibit an attractive air stable *n*-type performance with a moderate electron mobility of 0.15 cm^2^ V^−1^ s^−1^, a superior contrast of optimized traditional thin films, induced by highly ordered reassembly. Considering the structural diversity and the rearrangement ability, this solvent annealing process affords abundant room for exploring new functional organic small molecules nanowires. To further explore the potential application of SVA nanowires, fabricating *n*-type organic field-effect transistors (OFETs) arrays based on nanowire alignment or even the complementary circuit applications are still in progress. It is believed that such in situ reorganized processes will provide the potential to construct new small-molecule nanoelectronics.

## Supplementary Materials

The following are available online at http://www.mdpi.com/1996-1944/12/3/438/s1, Figure S1: AFM images of spin-coated morphology of PDI_8_-CN_2_ at a concentration of 5 mg/mL on Si/SiO_2_ substrate, Figure S2: Profile image across the white line in AFM images of PDI_8_-CN_2_ nanowires from 1:20 *w*/*w* solution on Si/SiO_2_, Figure S3: Optical microscope image of PDI_8_-CN_2_ nanostrutures from 1:50 *w*/*w* solution on Si/SiO_2_ substrate after the SVA process, Figure S4: XRD pattern of the prepared nanowires from the SVA treating PDI_8_-CN_2_/PMMA nanostructure, Figure S5: UV-vis absorption and photoluminescence spectra of PDI_8_-CN_2_ solution (black), nanowire (red), Figure S6: Confocal fluorescence microscope images of spin-coated PDI_8_-CN_2_ nanostructures of 0.3 mg/mL 1,2-dichlorobenzene solution on Si/SiO_2_ substrate, Figure S7: Optical microscope images of spin-coated pure PDI_8_-CN_2_ system with different concentrations in 1,2-dichlorobenzene on Si/SiO_2_ substrate before and after SVA: (a–c) as-cast; (d–f) after SVA; (a,d) 0.3 mg/mL; (b,e) 1 mg/mL; (c,f) 2 mg/mL. Different scale bars are used for comparison.
