# Supplementary material for: Investigation of Self-Assembly and Charge-Transport Property of One-dimensional PDI8-CN2 Nanowires by Solvent-Vapor Annealing
Source: Materials (Basel). 2019 Jan 31;12(3):438. doi: 10.3390/ma12030438 (PMC6384653; doi:10.3390/ma12030438)
Supplement: Supplementary file 1 [file materials-12-00438-s001.pdf]

materials

Supplementary Materilas: Investigation of Self-Assembly and Charge-Transport Property of One-dimensional PDI_8-CN_2 Nanowires by Solvent Vapor Annealing

Haixiao Xu, Jianqun Jin, Jing Zhang, Peng Sheng, Yu Li, Mingdong Yi and Wei Huang

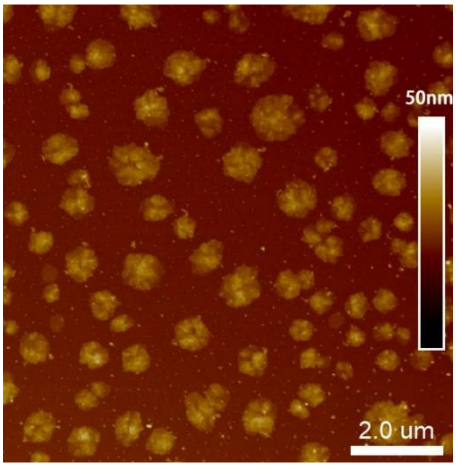

Figure S1. AFM images of spin-coated morphology of PDI_8-CN_2 at a concentration of 5mg/ml on Si/SiO_2 substrate.

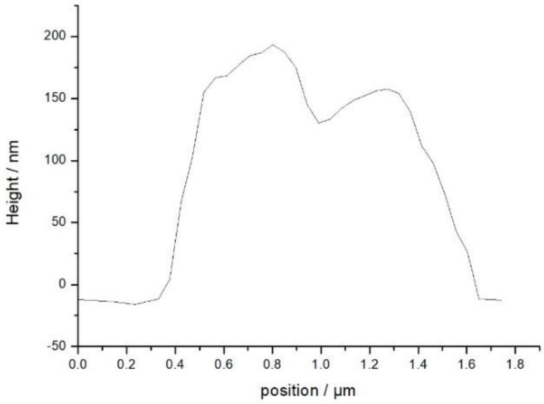

Figure S2. Profile image across the white line in AFM images of PDI_8-CN_2 nanowires from 1:20 w/w solution on Si/SiO_2.

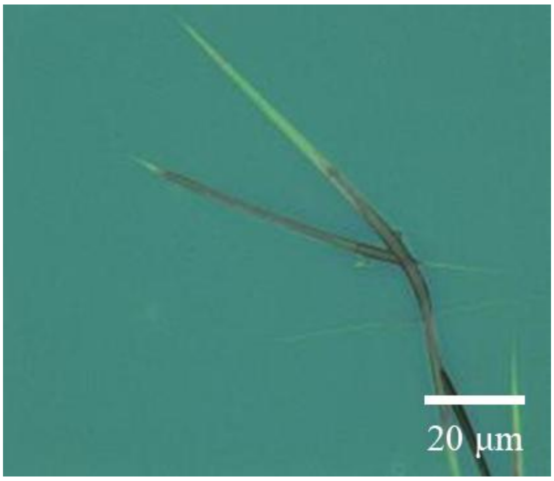

Figure S3. Optical microscope image of PDI8-CN_2 nanostrutures from 1:50 w/w solution on Si/SiO_2 substrate after the SVA process.

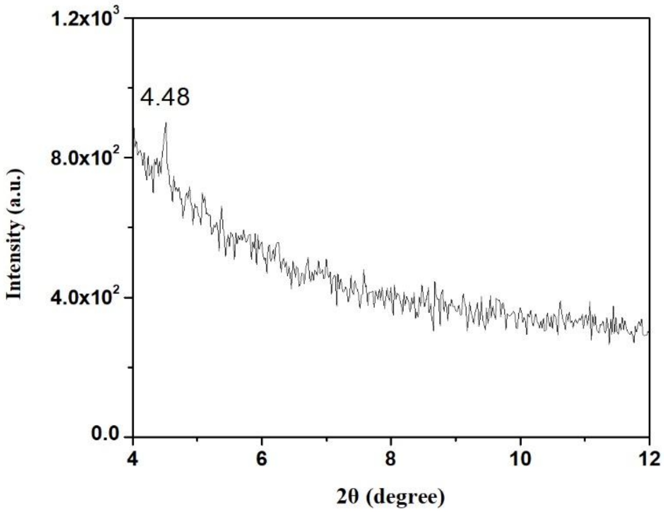

Figure S4. XRD pattern of the prepared nanowires from the SVA treating PDI8-CN_2/PMMA. nanostructure.

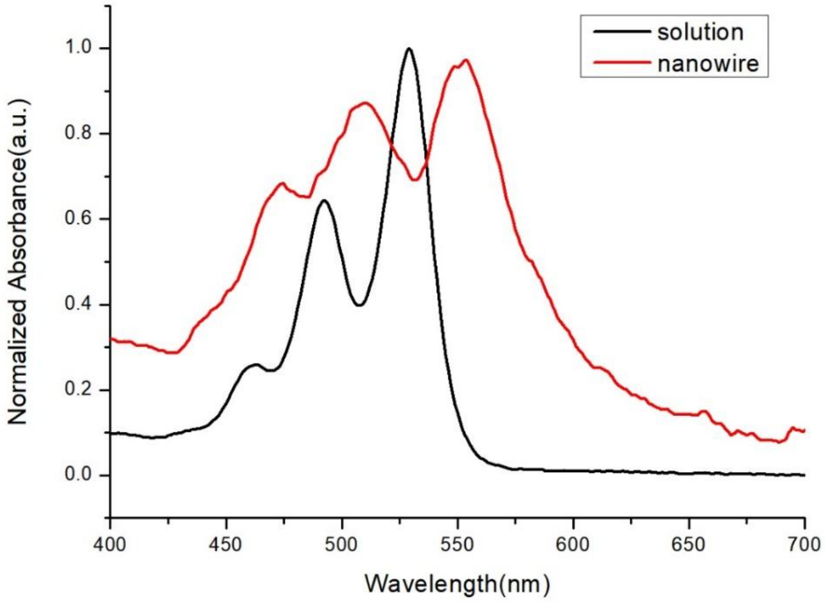

Figure S5. UV−vis absorption and photoluminescence spectra of PDI_8-CN_2 solution (black), nanowire (red).

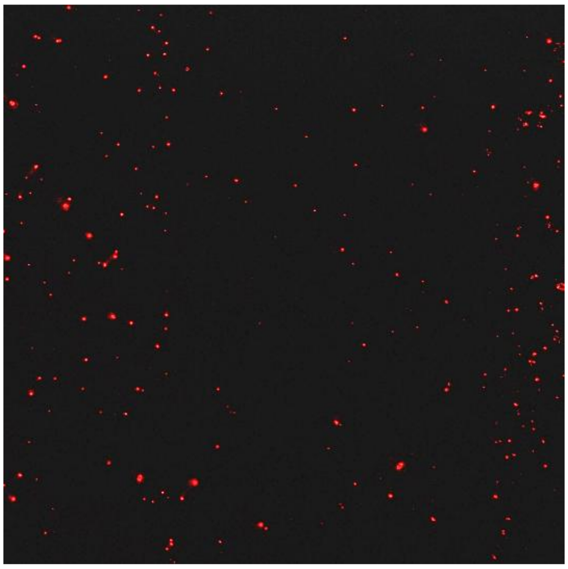

Figure S6. Confocal fluorescence microscope images of spin-coated PDI_8-CN_2 nanostructures of 0.3mg/ml 1,2-dichlorobenzene solution on Si/SiO_2 substrate.

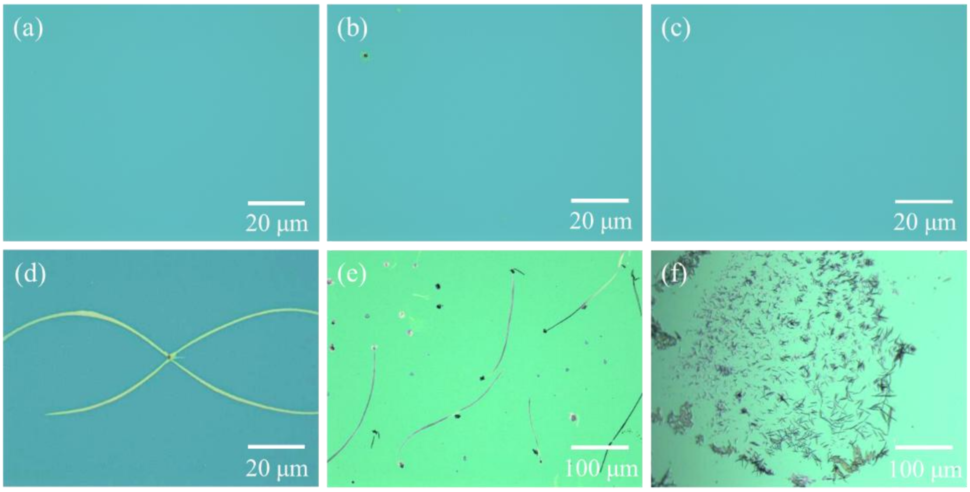

Figure S7. Optical microscope images of spin-coated pure PDI_8-CN_2 system with different concentrations in 1,2-dichlorobenzene on Si/SiO_2 substrate before and after SVA: (**a**–**c**) as-cast; (**d**–**f**) after SVA; (**a**,**d**) 0.3 mg/ml; (**b**,**e**) 1mg/ml; (**c**,**f**) 2mg/ml. Different scale bars are used for comparison.

© 2019 by the authors. Submitted for possible open access publication under the terms and conditions of the Creative Commons Attribution (CC BY) license (http://creativecommons.org/licenses/by/4.0/).
